# Evaluation of the Strontium Isotope Ratios in Soil–Plant–Fruit: A Comprehensive Study on Vignola Cherry (Ciliegia di Vignola PGI)

**DOI:** 10.3390/foods14091492

**Published:** 2025-04-24

**Authors:** Lisa Lancellotti, Veronica D’Eusanio, Daniela Manzini, Caterina Durante, Andrea Marchetti, Lorenzo Tassi

**Affiliations:** 1 Centro Interdipartimentale Grandi Strumenti (CIGS), University of Modena and Reggio Emilia, Via G. Campi 213/A, 41125 Modena, Italy; lisa.lancellotti@unimore.it (L.L.); daniela.manzini@unimore.it (D.M.); 2 Department of Chemical and Geological Sciences, University of Modena and Reggio Emilia, Via G. Campi 103, 41125 Modena, Italy; veronica.deusanio@unimore.it (V.D.); caterina.durante@unimore.it (C.D.); lorenzo.tassi@unimore.it (L.T.); 3 National Interuniversity Consortium of Materials Science and Technology (INSTM), 50121 Florence, Italy

**Keywords:** mass dependent fractionation, MC-HR-ICP/MS, strontium isotope ratios, Vignola PGI Cherry, geographic traceability

## Abstract

This study investigates the potential of strontium isotopes as a geographical tracer for Vignola cherries. Despite several studies having employed this indicator to trace the origin of food products, the mechanisms underlying the fractionation and translocation of strontium from soil to edible parts remain poorly understood. In this study, the ^91^Zr/^90^Zr ratio was used as a normalization pair to correct measurements of ^87^Sr/^86^Sr and ^88^Sr/^86^Sr (δ^88^Sr). Soil, cherry branches, and fruit samples were collected from various producers and locations. Isotopic analyses were carried out using a double-focusing multi-collector–inductively coupled plasma/mass spectrometer (MC-ICP/MS). External correction was applied using the ^91^Zr/^90^Zr ratio, assuming both equal and different fractionation factors for Sr and Zr isotopes. Results from both correction models showed improved accuracy by accounting for fluctuations in instrumental mass bias. Regarding the translocation of strontium, the data indicate an increase in ^88^Sr of approximately 0.2‰ from soil to plant tissue. This trend was consistent across all sampled locations.

## 1. Introduction

The growing interest in food traceability, along with increasing demands for stricter legal protections throughout the production chain, has led to a surge in scientific publications exploring various analytical methods to ensure product authenticity and regulatory compliance [1]. A review of the literature available in major scientific databases (PubMed, Web of Science, and Scopus) indicates that, over the past decade, more than 18,000 academic papers have been published on products bearing Geographical Indication labels, mainly Protected Designation of Origin (PDO) and Protected Geographical Indication (PGI). These studies have significantly advanced the understanding of food provenance and highlighted opportunities for innovation and quality enhancement within production chains. Despite the development and implementation of various analytical strategies, no universal method has yet been established. The selection of an appropriate analytical technique strictly depends on the specific food matrix and the characteristics of its production process. Broadly, these analytical approaches can be classified as either targeted or non-targeted [2,3,4]. Targeted approaches focus on a predefined set of known chemical parameters, enabling precise quantification and direct comparisons. In contrast, non-targeted methods [5] rely on comprehensive profiling techniques to generate an analytical fingerprint of the food product, allowing for the detection of subtle compositional variations. In targeted analyses, the selected indicators can be further categorized into primary and secondary indicators. Primary indicators, such as elemental and isotopic composition, are directly linked to the environmental and geological features of a specific region, thus providing strong evidence for geographical origin. Secondary indicators, including volatile compounds, organic acids, and sugars, are more influenced by the production process itself and often require extensive characterization of the food matrix to be effectively interpreted [6,7].

Among the various isotopic systematics, the strontium isotope ratio (^87^Sr/^86^Sr) is a well-established bio-geochemical tracer that has been widely applied across multiple research fields, including biology–ecology [8,9,10], geology and archaeology [11], and food science [12,13]. In the context of food authentication, the radiogenic ^87^Sr/^86^Sr ratio has shown significant promise, supporting the development of reliable models for verifying product authenticity and establishing geographical traceability. Successful applications have been reported for a wide range of food matrices, including plant-derived products such as wine [6,14,15,16,17,18], oil [19,20], asparagus and pistachio [21], hazelnut [22], and wheat [23], as well as animal-derived foods, including meat [24,25], fish [26], milk, and dairy products [25,27]. The main advantage of the ^87^Sr/^86^Sr system lies in its strong geological dependence, as strontium in plants and animals is primarily sourced from the local environment, and its isotopic signature is not significantly altered by metabolic processes. However, some limitations should also be acknowledged. The isotopic composition of strontium can be influenced by complex factors such as water availability, irrigation sources, or geological heterogeneity within a region, which may require careful sampling strategies and detailed geological background knowledge. Nonetheless, the ^87^Sr/^86^Sr ratio remains a powerful tool for enhancing the transparency and reliability of food provenance systems.

Strontium has four isotopes: ^84^Sr, ^86^Sr, ^87^Sr, and ^88^Sr. Among these, ^87^Sr is radiogenic, meaning its relative abundance increases due to the radioactive decay of ^87^Rb. This transformation occurs through beta decay, with a half-life constant of 4.92 × 10^10^ years, which is approximately ten times the estimated age of the Earth [28]. The use of ^87^Sr/^86^Sr isotope ratios for food traceability relies on the fact that this ratio varies naturally in the Earth’s crust as a result of differences in the relative concentrations of rubidium and strontium (Rb/Sr), the half-life constant, and the time elapsed, as described by the radioactive decay equation [28]. Furthermore, in soils, bioavailable strontium can be absorbed by plant roots and transported through conducting tissues to various plant organs, including fruits. Importantly, during this translocation process, the ^87^Sr/^86^Sr ratio remains almost unchanged, as isotopic fractionation has a minimal effect on heavier elements. Therefore, the strontium isotopic signature of a food product can directly reflect the geological characteristics of its growing environment. However, measurable and meaningful variations in the isotopic ratios of heavy elements can occur from chemical, biological, and physical processes [29]. These variations follow a mass-dependent fractionation mechanism, meaning that the isotopic composition of natural samples may be influenced by physicochemical transformations occurring throughout their environmental or biological history [29,30].

The accuracy of isotope ratio measurements must be ensured using internationally recognized isotopically certified reference materials, which account for mass discrimination effects arising from sample composition and/or instrumental determination. In the case of strontium, instrumental mass bias encountered during inductively coupled plasma/mass spectrometry (ICP/MS) element isotope measurements, particularly when determining the ^87^Sr/^86^Sr ratio, is generally corrected using the ^88^Sr/^86^Sr ratio as an internal normalizing isotopic couple. This ratio is generally assumed to remain constant in nature (^86^Sr/^88^Sr = 0.1194). This approach effectively removes any mass-dependent fractionation effects, whether natural and/or instrumental, thereby isolating the radiogenic contribution of ^87^Sr. The assumption of a constant ^86^Sr/^88^Sr ratio does not affect the feasibility of using the radiogenic ^87^Sr/^86^Sr ratio for tracing the provenance of environmental and food, as well as for studying human and animal migration. However, the isotopic composition of Sr isotopes, such as ^86^Sr and ^88^Sr, may vary due to mass dependent isotopic fractionation through various physicochemical processes in nature [30,31,32,33,34]. Investigating these processes may provide additional information regarding the mechanisms of nutrient uptake during element translocation from soil to plant/fruit and ultimately to the final product. Therefore, for a more thorough investigation of samples and processes, the assumption of a constant ^88^Sr/^86^Sr ratio must be reconsidered. In this study, to overcome the limitation of assuming a constant ^88^Sr/^86^Sr ratio for mass bias correction, a different isotopic ion couple, ^91^Zr/^90^Zr, was employed [33,35,36], and the correction procedure for determining ^87^Sr/^86^Sr and ^88^Sr/^86^Sr is discussed as well as the limiting factors of its application.

Based on these findings, this geographical tracer was used in this study as a preliminary attempt to establish an objective link between one of the typical products of the province of Modena, the ciliegia di Vignola PGI (Vignola PGI cherry), and its territory of origin.

Vignola PGI cherry is a food of undoubted interest both for its nutraceutical properties and because it was granted the Protected Geographical Indication designation by the European Union in 2012 [37]. Its production area spans approximately 4.5 km^2^ and encompasses 28 municipalities across the provinces of Modena and Bologna [http://www.consorziociliegiadivignolaigp.it “URL (accessed on 22 April 2025)”]. The presence of the Vignola PGI cherry has led to an important commercial, social, and economic impact on the area of Modena districts, resulting in a significant increase in the number of farms, the establishment of processing and marketing cooperatives, and the growth of the fruit’s production and commercial distribution. This fruit is rightfully categorized as a functional food thanks to its rich nutritional content, including minerals, vitamins, polyphenols, and anthocyanins [38]. This product is strongly linked to the territory, with its production standards defined by a set of regulations that outline various criteria, including varietal specifications, quality characteristics, production area, fertilization and pruning practices, phytosanitary treatments, harvesting methods, packaging, and labelling. Although several studies have focused on the characterization of the Vignola cherry [38,39], to the best of our knowledge, this is the first instance where isotopic approaches have been applied to support the connection between ciliegia di Vignola PGI and its geographic origin. Therefore, to achieve the objectives of this research, the first step was to conduct a pilot study in which the ^87^Sr/^86^Sr isotopic ratio was analyzed in soil, cherry tree leaves, and the corresponding fruits from two farms, namely, A (Castelvetro di Modena) and B (Vignola). These two farms were selected due to their accessibility, as well as their contrasting geographical locations, one situated in the hills and the other in the plains. In total, 100 samples of Vignola PGI cherries, kindly provided by the Consortium for the Protection of the Vignola PGI cherry, were analyzed, and their isotopic ratios were compared with those of soils in the Modena province from previous traceability studies.

## 2. Materials and Methods

### 2.1. Reagents and Materials

Ultrapure 65% nitric acid (HNO_3_) was prepared in-house from analytical-grade stock solution (Carlo Erba, Milan, Italy) using a SAVILLEX DST 1000 sub-boiling system (Savillex Corp., Eden Prairie, MN, USA). Ultrapure water (ASTM TYPE I) was obtained with a Millipore IQ 7000 system equipped with an IQ Element module (Millipore, Milan, Italy). A 1 M Suprapur-grade ammonium nitrate (NH_4_NO_3_) was employed as the extraction eluent. Zirconium nitrate (Zr(NO_3_)_4_), of analytical grade purity, was used for mass bias correction. Both reagents were purchased from Merck Millipore (Milan, Italy). For calibration and quality control, the certified reference material SrCO_3_, NIST SRM 987 (NIST, Gaithersburg, MD, USA), was used. This standard has a certified ^87^Sr/^86^Sr isotopic ratio of 0.71034 ± 0.00026 (the uncertainty “u” is expressed as twice the standard deviation, 2 sd, u = 2 sd) [40] and a generally accepted ratio of 0.710260 ± 0.000016 (u = 2 sd) [41], serving as a benchmark for the bracketing procedure and accuracy assessment.

The ICP-multielement solution IV-ICP-MS-71A, used for Sr and Rb concentration determination, was obtained from Inorganic Ventures (Christiansburg, VA, USA).

The Eichrom SR-B100-S (50–100 μm) Sr resin, used for Sr/Rb separation, was purchased from Eichrom Technologies Inc. (Lisle, IL, USA). All containers and vessels made of perfluoroalkoxy (PFA) used in the preparation of samples and solutions underwent a rigorous cleaning protocol, consisting of a wash with 10% sub-boiled nitric acid at elevated temperature, followed by multiple rinses with ultrapure Milli-Q water to eliminate potential contaminants. Sample and standard solution preparation was carried out using gravimetric techniques, employing a Mettler AE200 analytical balance (Mettler Toledo S.p.A., Milan, Italy) with a precision of 0.0001 g. To ensure minimal contamination during all stages of the analytical workflow, procedures were performed in a cleanroom environment equipped with HEPA-filtered air systems.

### 2.2. Instrumentation

Cherry branch samples were finely ground using a Fritch Pulverisette 14 model centrifugal mill (ECO Scientifica, Milan, Italy), fitted with a 12-rib rotor made of pure titanium, a 250 μm titanium sieve ring featuring trapezoidal apertures, and a Teflon-coated collection container. The milling process was carried out at a rotor speed of 16,000 rpm.

Soil samples were processed using a PM100 planetary ball mill (Retsch, FKV, Bergamo, Italy) equipped with a single 250 mL agata grinding jar and two milling balls. Specific milling parameters are detailed in Table S2 (reported as Supplementary Materials). The milling procedure involved two grinding cycles, targeting only the coarse fraction (>250 μm) for the second round. For particle size separation, an AS200 vibratory sieve shaker (Retsch; FKV, Bergamo, Italy) was employed, using a stainless steel sieving stack comprising 315 μm and 250 μm mesh sieves and a bottom collector for the <250 μm fraction. Sieving was performed under dry conditions, with an amplitude setting of 70% and a total run time of 5 min, using a throwing action to achieve effective fractionation.

Acid digestion of ground cherry branches and dried cherry fruits was carried out using a microwave digestion system (Ultrawave, Milestone, Bergamo, Italy), featuring a five-position holder compatible with 40 mL quartz vessels. This system operates with a single high-pressure, high-temperature chamber, enabling the simultaneous treatment of multiple samples. Batches of five were processed, with each digestion performed on approximately 5 g of dried plant material.

The system’s 1500 W magnetron was regulated automatically by the integrated Ultrawave software program (Milestone easyControl 640, Rev. 01-R4, 2011, Bergamo, Italy), which maintained the heating profile according to theoretical parameters defined in the Supplementary Materials. Digestion consistently yielded transparent solutions with no visible particulates.

Subsequent quantification of Sr and Rb content in the digests was conducted via inductively coupled plasma mass spectrometry, employing a quadrupole-based instrument (ICP/qMS; XSeries II, Thermo Fisher Scientific, Bremen, Germany). Detailed operating parameters for both the digestion and ICP/qMS analysis can be found in Tables S3 and S4 (see Supplementary Materials).

The instrument’s 1500 W magnetron power was automatically managed by Ultrawave control software, ensuring a heating ramp aligned with the theoretical trend based on preset parameters (reported in the Supplementary Materials). All digested solutions were clear, with no visible residue.

The Sr and Rb concentrations in all samples were determined using an inductively coupled plasma interfaced to a quadrupolar mass analyzer (ICP/qMS; XSeries II model, Thermo Fisher Scientific, Bremen, Germany). The instrumental parameters and experimental setup conditions are reported in Tables S3 and S4 (reported as Supplementary Materials) for the microwave technique (MW) and ICP/qMS instruments, respectively.

Strontium isotope ratios (^87^Sr/^86^Sr and ^88^Sr/^86^Sr) and zirconium isotope ratio (^91^Zr/^90^Zr) were determined using a double-focusing multi-collector–inductively coupled plasma/mass spectrometer, (MC-ICP/MS; Neptune, Thermo Fisher Scientific, Bremen, Germany). A cyclonic-Scott nebulizer chamber with an auto-aspirating nebulizer device was used for Sr isotope measurements in solutions with Sr concentration between 30 to 200 μg kg^−1^. For samples with Sr concentrations below 30 μg kg^−1^, an Apex IR high-sensitivity desolvating nebulizer (Elemental Scientific Inc., Omaha, NE, USA) was used. Therefore, in this study, the former device was used for Sr and Zr isotope measurements in soil and branch samples, while the latter introduction system was used for the same elements in the cherry solutions. The instrumental parameters are reported in Table S5 (reported as Supplementary Materials).

The plasma positioning/condition and ion lens setting were tuned daily for maximum sensitivity and optimal flat-topped peak shape signal.

Table S5 (reported as Supplementary Materials) shows the cup configuration used for the determinations, which allows for the collection of all the isotopes required for interference and mass bias correction. Data were acquired in static and low-resolution mode. An initial baseline correction and peak-centering procedure were performed before starting each sample/standard/blank reading procedure to correct for short-term instrument drift.

To monitor potential instrumental drift, measurements were conducted using a bracketing sequence structured as [blank/sample/blank/standard/blank]. The mean signal intensity obtained from the bracketing blanks (prepared in 4% *w*/*w* HNO_3_) was subtracted from the corresponding sample or standard readings. Following this correction, Sr/Zr isotope ratios were determined using established computational protocols that account for both isobaric interferences and instrumental mass bias effects.

### 2.3. Sampling and Samples Pretreatment

To characterize the geographical origin of Vignola PGI cherries, different matrices were analyzed for their ^87^Sr/^86^Sr and ^88^Sr/^86^Sr ratios. Soils, cherry branches, and cherries were collected from two different locations within the production district in the province of Modena: A (Castelvetro di Modena) and B (Vignola). Cherry branches were specifically chosen for sampling due to the advantages of the “active” uptake process performed by plant roots, which directly access the reservoir of bioavailable elements in the soil. This mechanism extends the sampling influence to a broader area surrounding the plant’s growth site. Consequently, the measured ^87^Sr/^86^Sr and ^88^Sr/^86^Sr ratios reflect the combined effects of multiple processes, including weathering reactions, contamination from fertilizers, and atmospheric deposition through aerosol uptake, among others [42]. Plants serve as effective bioindicator for geochemical investigations due to their capacity to selectively absorb and accumulate the bioavailable fraction of elements present in the soil. This approach enables a more representative sampling over a broader soil profile and offers a natural integration of elemental uptake processes occurring over varying spatial and temporal scales.

#### 2.3.1. Soil Sampling

As detailed in Table S1 (Supplementary Materials), five soil samples were obtained from each plot using a handheld percussion auger with a 3 cm internal diameter, specifically designed to minimize sample disturbance. This method allowed for the collection of continuous soil profiles reaching depths between 60 and 80 cm. The uppermost 10 cm (0–10 cm) were discarded to eliminate potential contamination from grass or surface debris.

Sampling was conducted during the spring season, at the beginning of the plant’s vegetative phase. After sampling, the soil samples were manually disaggregated, and extraneous materials such as rocks and plant residues were meticulously removed to ensure the integrity of the analytical portion of the sample. Each sample was then finely minced using a Teflon spatula and subjected to the following pre-treatment steps: 50 g of soil was dried at 105 °C for 48 h, ground into a fine powder using a planetary mill, and subsequently stored in hermetically sealed polystyrene containers.

#### 2.3.2. Branches and Cherries Sampling

Simultaneously with soil sampling at sites A and B, cherry tree branches were gathered from plants located near the soil extraction points. Each branch, approximately 10 to 20 cm in length, was selected to match the number of soil samples collected. The plant materials were oven-dried at 105 °C for 48 h, then sectioned into 1 cm fragments, placed in polystyrene containers, and stored at ambient conditions until analysis.

Cherry samples were collected from both site A and B, with 5 samples obtained from each site.

The sampling strategy was developed in close alignment with geographical production area (Modena and Bologna districts, Italy) and the seasonal distribution of the cultivars. Indeed, Vignola IGP cherries include a wide range of cultivars with three distinct ripening periods. In particular, harvesting occurs over an extended period, typically from 15–20 May (early varieties) to 10–15 July (late varieties). Cherries were sampled from 40 producers across the ciliegia di Vignola PGI production area, covering the entire harvest period from May to July, resulting in a total of 100 cherry samples. From a geographical point of view, the production area for Vignola cherries overlaps substantially with that of a previous study [43]. The coherence in sample size reflects our strategy to ensure spatial resolution sufficient to distinguish territorial isotopic signatures, as demonstrated successfully in the wine study [43]. To extend their shelf life, the cherries were pitted, and the pulp was dried in an oven at 105 °C for 24 h. Therefore, a total of 130 samples (10 soils, 10 cherry branches, 10 cherry samples from sites A and B, and 100 cherries from the whole production area) were processed.

### 2.4. Experimental—Sample Processing

#### 2.4.1. Soil Samples

Separate portions of the same air-dried soil samples were pulverized using a planetary ball mill, following the procedure outlined above. To isolate the bioavailable strontium fraction, extractions were carried out with a 1 M ammonium nitrate (NH_4_NO_3_) solution, in accordance with the DIN 19730 protocol [44]. The resulting extract was passed through a 0.2 μm cellulose acetate membrane filter and transferred into 30 mL PFA containers. Before proceeding with Sr/Rb separation, total strontium concentrations were determined, and samples were diluted with 8 M nitric acid to reach an approximate Sr concentration of 200 μg kg^−1^.

#### 2.4.2. Cherry Branches and Cherries

Microwave-assisted mineralization was performed on cherry branch and pulp samples. Approximately 1 g of each sample was accurately weighed into quartz vessels suitable for microwave digestion, followed by the addition of 7 mL of 65% HNO_3_ and 3 mL of deionized water. The digestion process consistently yielded clear or slightly yellow solutions, which were subsequently transferred into PFA containers for storage. To minimize any risk of cross-contamination, each digestion cycle was followed by a cleaning protocol involving 6 mL of 65% HNO_3_ and 4 mL of ultrapure Milli-Q water, using the same microwave program. Prior to the Sr/Rb separation step, total strontium content was quantified to identify the appropriate dilution needed to achieve target concentrations of ~200 μg kg^−1^ for branch samples and ~20 μg kg^−1^ for cherry pulp.

The repeatability and reproducibility of the sample preparation procedures were monitored by introducing three control samples (soil, cherry branch, and cherry) during sample preparation. The calculated uncertainties, relative to the mean values of the measured isotope ratios, ^87^Sr/^86^Sr and ^88^Sr/^86^Sr, were associated with all respective samples. At the same time, repeatability and reproducibility of isotopic ratio data were monitored using the NIST SRM 987 standard measured under different operating conditions, i.e., by conventional nebulization and with the Apex IR apparatus. Table S6 (reported as Supplementary Materials) reports the mean values obtained for the NIST SRM 987.

All average values fall within the range of the NIST-certified value (0.71034 ± 0.00026) and demonstrate excellent agreement with the commonly accepted high-precision value of 0.71026 ± 0.00002 (u = 2 sd) [41]. Higher uncertainties were observed when measurements were performed using the APEX IR setup at very low concentrations (0.71028 ± 0.00006), as shown in Table S6 (reported as Supplementary Materials). This increased uncertainty is likely due to the lower signal intensities recorded by the Faraday cups.

#### 2.4.3. Removing of the Isobaric Interferences: Sr/Rb Separation

To mitigate interferences and matrix effects on Sr isotopic measurements, a matrix separation procedure using specific cation exchange resins was applied. Briefly, sample solutions in 8 M HNO_3_ were loaded onto SPE columns packed with Eichrom Sr-specific resin, which tends to retain Sr^2+^ over other cations under highly acidic conditions [45]. After washing with 8 M HNO_3_ to elute all the metals with low affinity for the resin, Sr was quantitatively recovered by eluting the SPE with a 2% *v*/*v* HNO_3_ solution [45]. This procedure is widely consolidated within the literature and has been extensively validated by our research group in previous work, where recovery rates and quality control parameters are reported in detail [46].

#### 2.4.4. Delta Notation

The ^88^Sr/^86^Sr values are generally reported in the literature using the per mil delta notation, δ^88^Sr‰, relative to an international standard, according to the following general equation (Equation (1)) [47]:

(1) $$\delta R‰=[\left(\frac{R_{s}}{R_{std}}\right)-1]\times 1000$$

where *R* = ^H^F/^L^F, with F the fractional abundance of the heavy, ^H^F, and *light*, ^L^F, isotope (namely, ^88^Sr for ^H^F and ^86^Sr for ^L^F, respectively). *R_s_* is the respective isotope number ratio of a sample, while *R_std_* is that of the standard. In this study, *R_std_* was set equal to 8.37861 ± 0.00325 (u = 2 s) [40].

#### 2.4.5. Mass Bias Correction

Given that the assumption of a constant ^88^Sr/^86^Sr ratio does not account for potential mass-dependent fractionation effects during the translocation of elements between soil, plant, and fruit, an alternative methodological approach was employed.

**Figure 1:**
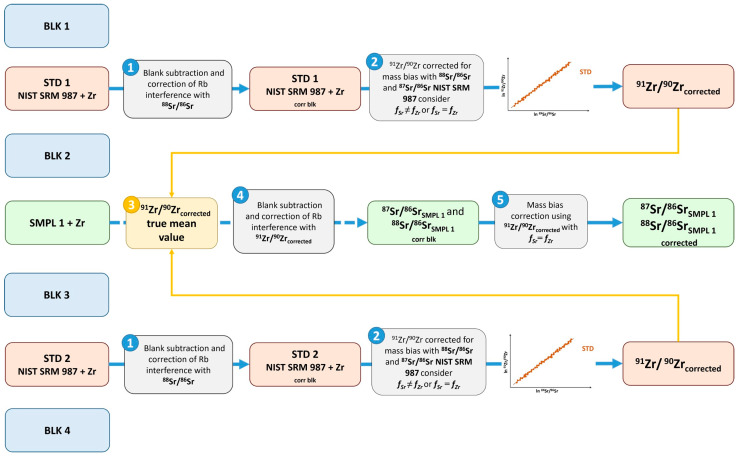
Flow chart of the analytical procedure adopted for the external correction of the mass bias for the ^87^Sr/^86^Sr and ^88^Sr/^86^Sr in soil, plant, and cherry samples. Numbers represent the procedure steps.

This approach involved both internal and external correction procedures using an exponential model for instrumental mass bias compensation. Figure 1 provides an overview of the procedure, highlighting the main steps following a blank/standard/sample bracketing approach for the measurements.

The reading process starts with the measurement of a blank solution, Blk 1, 4% ultrapure nitric acid in ultrapure water. (step 1 of Figure 1) The faraday cups readings, see Table S5, subsequently averaged with the values of Blk 2, are subtracted from the values measured for standard solution STD 1. This procedure is aimed at calculating the isotopic ratio of Zr using the NIST 987 standard for the instrumental mass bias correction using the exponential correction model, f_Sr_ = f_Zr_, and/or the regression method, f_Sr_, different from f_Zr_ (step 2 of Figure 1). In the latter case, the regression model can be applied only offline, that is, after having collected the data of all the standards programmed in the sequence, STD 1–STD n, for the construction of the graph ln ^91^Zr/^90^Zr vs. ln ^88^Sr/^86^Sr or ln ^91^Zr/^90^Zr vs. ln ^87^Sr/^86^Sr.

The true ^91^Zr/^90^Zr isotope ratios were calculated using the certified data of 0.71026 for ^87^Sr/^86^Sr and the IUPAC-reported value of 0.1194 for ^86^Sr/^88^Sr (the value conventionally accepted by the International Union of Geosciences (IUGS), Sub-commission on Geochronology [48]). These values were used as normalizing factors following an exponential law: step 3 of Figure 1.

The “corrected” ^91^Zr/^90^Zr ratios were used in the normalization of the data, steps 4 and 5 of Figure 1, for the calculation of the ^87^Sr/^86^Sr and δ^88^Sr ratios.

The external correction was performed using the corrected mean value of ^91^Zr/^90^Zr to normalize Sr ratios (^87^Sr/^86^Sr, and δ^88^Sr) during sample processing, in accordance with the Russell, Papanastassiou, and Tombrello exponential law [49].

Isobaric interferences due to ^86^Kr on ^86^Sr and ^87^Rb on ^87^Sr were corrected through blank subtraction for the former, while for the latter, the ^85^Rb ion was measured, and a ^87^Rb/^85^Rb ratio of 0.38567 was used to evaluate the ^87^Rb contribution. The ^87^Rb/^85^Rb ratio was corrected for instrumental mass bias using the same exponential law, employing the true ^88^Sr/^86^Sr ratio [29] for internal correction and the ^91^Zr/^90^Zr ratio for external correction.

## 3. Results and Discussion

### 3.1. Mass Bias Correction Procedure

The absence of a certified international reference standard for the ^91^Zr/^90^Zr ratio necessitates an initial step in mass bias correction for ^87^Sr/^86^Sr and ^88^Sr/^86^Sr measurements. This step involves determining the isotope ratio value of these two isotopes to serve as an external reference for instrumental mass bias normalization. For this purpose, NIST 987 SrCO_3_ is commonly used as a reference standard. The external correction method generally assumes that the fractionation coefficients for the involved isotopes are identical (f^analyte^/f^int.std^ ratio equal to 1). While this approximation is generally accepted for isotopes of the same element, some deviations may occur due to mass-dependent or mass-independent isotope fractionation processes [50]. To verify the presence of mass independent fractionation (MIF) process in Sr isotopes, a three-isotope plot of ln (^87^Sr/^86^Sr) vs. ln (^88^Sr/^86^Sr) was constructed using measurements of NIST 987 solutions in the presence of the Zr external standard (Figure S1, reported as Supplementary Materials). The obtained slope (0.5039 ± 0.0013, Slope ± sd) closely matches the theoretical value of 0.5036, derived from Equation (2), under the assumption of an exponential law for mass bias correction in ion transmission [51]:

(2) Slope(Sr87Sr88)=ln⁡(M87M86)ln⁡(M88M86)

where M_87_, M_86_, and M_88_ are the exact atomic masses of the three isotopes. Therefore, based on these results, it is possible to exclude the presence of the MIF phenomenon.

**Figure 2:**
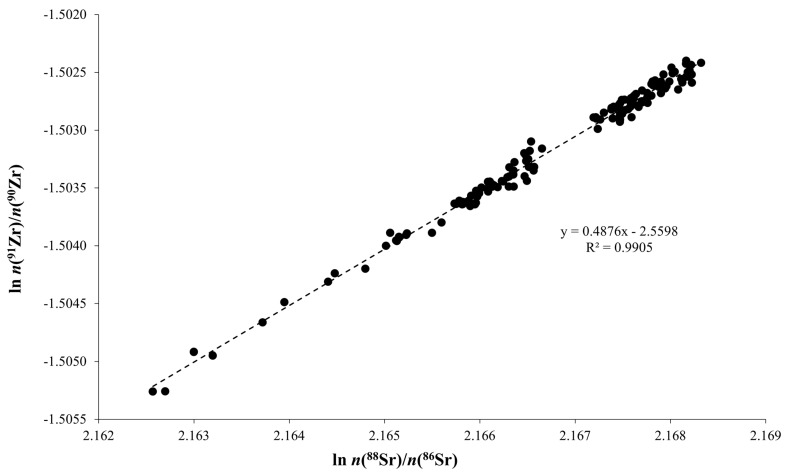
Three isotopes plot for the ln (^91^Zr/^90^Zr) vs. ln (^88^Sr/^86^Sr) measured on the NIST 987 solutions, spiked with the Zr(NO_3_)_4_ external standard, for the different working sessions. Theoretical slope for the investigated system, based on an exponential correction law, is obtained by ^91^Zr/^90^Zr = ln (M91/M90)/ln (Mxx/M86). Comparison between measured and theoretical slopes is reported in Table 1.

Now, when Zr is used to correct Sr ratios, assuming identical mass bias correction factors for Sr and Zr may lead to inaccurate data [33]. In this case, an alternative method that utilizes external standardization for mass bias correction was employed [52,53,54]. This approach is based on a linear regression correlation between the logarithms of different isotopic ratios, providing several advantages over conventional standardization. Unlike the standardization method, which relies on using an isotopic Certified Reference Material to calibrate the external standard ratio, this “regression” method for mass bias correction offers several significant advantages, such as obtaining a priori freedom from the assumptions of f^analyte^ = f^int.std^ and the ability to correct for the matrix-induced mass bias [50,55,56]. Figure 2 reports the ln (^91^Zr/^90^Zr) vs. ln (^88^Sr/^86^Sr) plot obtained by measuring the NIST 987 solutions spiked with Zr. In addition, Table 1 reports the calculated regression parameters obtained for both the ln (^91^Zr/^90^Zr) vs. ln (^88^Sr/^86^Sr) and ln (^91^Zr/^90^Zr) vs. ln (^87^Sr/^86^Sr) approaches.

The calculated slope values closely align with the theoretical data for similar fractionation behavior between strontium and zirconium isotopes. Based on these data, it is now possible to determine the mass bias corrected ^91^Zr/^90^Zr ratio for the sample correction. Based on the previous considerations and regardless of the strontium ratio used, the mean value obtained was 0.21797 ± 0.00003 (average ± 2 sd) for the ^91^Zr/^90^Zr ratio.

Although the discussion of the real isotopic composition of Zr is beyond the scope of this study, the determined ^91^Zr/^90^Zr value aligns well with those reported in previous research [57,58].

Although this approach generally provides accurate isotopic ratio determinations, it has limitations in terms of precision. This is due to the reliance on linear regression to generate the slope and intercept, which are subsequently used to calculate the mass bias corrected analyte ratio. The uncertainty associated with this process can be reduced only by increasing the number of measurement sessions and conveniently tuning the instrumental parameters to obtain the magnitude variations of mass bias effect [56]. Moreover, achieving reliable results requires strict matrix matching between the standard and sample solutions.

For these reasons, the ^91^Zr/^90^Zr ratio was also verified by considering the f^Sr^/f^Zr^ ratio equal to one. In this case, a value of 0.21802 ± 0.00003 (average ± 2 sd) for the ^91^Zr/^90^Zr ratio was obtained. The close agreement between the results derived from the regression approach and those from the exponential law calculation aligns well with the trend of the data reported in Figure 2, where the fitted line’s slope closely matches the theoretical one. The main advantage of this latter approach, coupled with the sample/standard/sample bracketing (SSB) technique relies on the capability to account for irregular fluctuations in instrumental mass bias during the measurement. On the other hand, this type of mass bias correction has been effective only for closely matching Sr and Zr concentrations in the standard and sample, suggesting that matrix-induced mass bias cannot be fully compensated by this approach. Furthermore, the obtained results indicate that the extent of the mass fractionation processes depends on and/or is correlated in some way to the element concentrations within the solution. As previously reported by other authors [33,59], the higher the concentration in the standard/sample solution, the greater is the mass bias. Therefore, to achieve the highest accuracy and precision in isotope ratio determinations, near perfect analyte separation is required. This minimizes matrix effects and ensures that analyte concentrations in standard/sample solutions and dilute solutions are closely matched.

By establishing the external correction isotope ratio, it is now possible to determine the ^87^Sr/^86^Sr and ^88^Sr/^86^Sr ratios or δ^88^Sr in soil, shoot, and cherry samples without the assumption of a constant ^88^Sr/^86^Sr ratio.

### 3.2. Reproducibility of Measurements

To assess the reproducibility of the isotopic ratio measurements, multiple control samples were analyzed. The raw data were processed to evaluate the ^87^Sr/^86^Sr and δ^88^Sr mass bias corrected values using the external correction procedure based on the ^91^Zr/^90^Zr isotopic couple. Therefore, a soil, cherry branch, and cherry sample were included in each measurement session and processed following the entire analytical procedure. Table 2 reports the results obtained for each matrix, including ^87^Sr/^86^Sr, ^88^Sr/^86^Sr, and δ^88^Sr variables, along with their corresponding standard deviation. The reported standard deviation values confirm the high concordance degree for the replicated measurements, and each of these uncertainties was associated with all samples of the same type.

### 3.3. Soil, Branches and Cherry Samples

**Figure 3:**
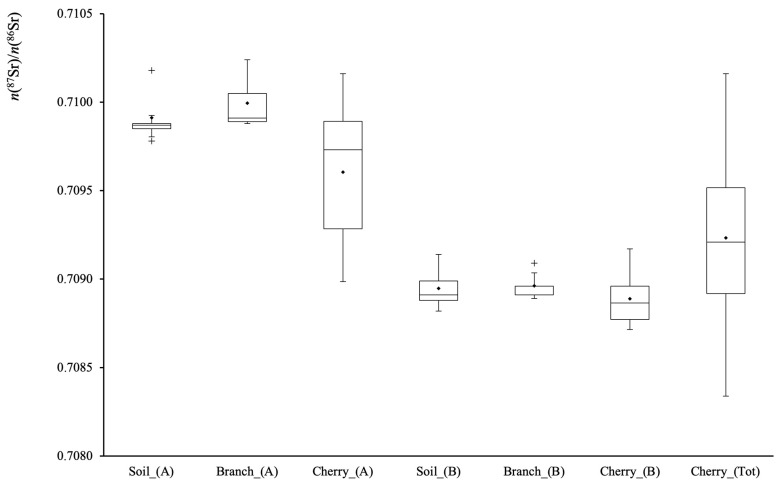
Box and whisker plot representation of the ^87^Sr/^86^Sr data measured for the soil, tree branches, and cherry matrices sampled from producer’ sites A (Castelvetro) and B (Vignola). Cherry_(Tot) represents the cherry distribution of the Vignola PGI cherry area. (+) symbol indicates sample outliers.

**Figure 4:**
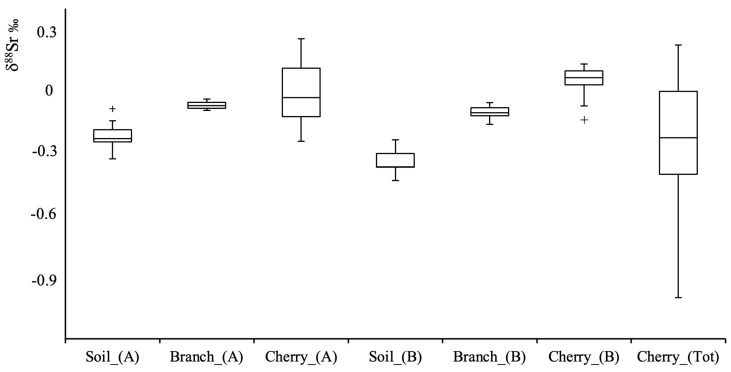
Box and whisker plot representation of the δ^88^Sr‰ data measured for the soil, tree branches, and cherry matrices sampled from producers A (Castelvetro) and B (Vignola). Cherry_(Tot) represents the cherry distribution of the Vignola PGI cherry area. (+) symbol indicates sample outliers.

In this study, the ^87^Sr/^86^Sr and ^88^Sr/^86^Sr ratios were analyzed along the cherry production chain, i.e., soil, tree branches, and cherry. The obtained ^87^Sr/^86^Sr and δ^88^Sr values, corrected for the instrumental mass bias using the external ^91^Zr/^90^Zr ratio, are reported in Table S7 and Table S8, respectively (reported as Supplementary Materials) and in graphical form, by box and whiskers plots, in Figure 3 and Figure 4, respectively.

Several key observations emerge from these findings. Firstly, the ^87^Sr/^86^Sr distribution shows a strong correlation between soil–branches–cherry at both sampling sites: A (Castelvetro) and B (Vignola). This agreement confirms that the isotopic signature of cherries accurately reflects the bioavailable Sr fraction absorbed by the plant roots, and vice versa. Moreover, in site A, the cherries data exhibit a broader isotopic distribution compared to the soil and branches. This could be due to variations in soil composition within the hilly area. Unfortunately, there is no confirmation that the analyzed cherries were collected from the same tree as the branches, which could contribute to this variability. Nonetheless, the measured isotopic ratios align well with the isotopic map obtained in a previous investigation on the Lambrusco wine production chain in the same Modena district [43]. It is worth noting that the previous investigation employed internal correction of Sr for correcting instrumental mass bias. Nevertheless, the results obtained for both fields A and B are consistent with the values determined in the “Lambrusco wine area of production” study, thus confirming the reliability of ^87^Sr/^86^Sr as a geographical traceability marker.

At the Vignola site (B), located near the Panaro riverbank, the sampled soils display the highest uniformity in ^87^Sr/^86^Sr ratios. Both the mean value and the low intra-field variability align with the isotopic map [43], which predicts homogeneous isotopic ratios along the banks of the Panaro river in the surroundings of Vignola.

The ^87^Sr/^86^Sr value confirms to be an excellent traceability marker in this case. In fact, the mean values of the soils and branches are comparable for both Castelvetro (t(9) = 0.70, *p* < 0.05) and Vignola sites (t(8) = 2.33, *p* < 0.05). Since the branch samples were obtained by mixing branches from the five closest trees to each soil sampling point and each tree acts as an “active sampler” of the soil via its root system, this consistency confirms that branches effectively reflect the average ^87^Sr/^86^Sr values of the surrounding soils.

Regarding the δ^88^Sr variable, the data distribution (Figure 4) for each investigated matrix shows an interesting trend. In fact, despite being influenced by the chosen reference ratio, the transition from soil to cherries indicates a fractionation mechanism during strontium uptake, leading to an enrichment of the heavier δ^88^Sr isotope along the food chain. Explaining this fractionation is challenging due to the multiple sources contributing to the plant’s bioavailable strontium, including fertilizers, atmospheric deposition, and chemical weathering. These factors significantly influence δ^88^Sr variability in plants, complicating the accurate determination of fractionation effects.

Furthermore, studies on plant uptake suggest a gradual reduction of δ^88^Sr from roots to leaves (or flowers) [60]. For example, Choi et al. [34] provided evidence that bean sprouts preferentially absorbed the lighter Sr isotope during the plant uptake. However, it is important to note that this fractionation may also result from preferential transport of distinct isotopes through different plant organs. Investigations associating calcium/strontium (Ca/Sr) ratios with δ^88^Sr suggest a preferential enrichment of ^88^Sr over ^86^Sr during translocation in plants [61]. This phenomenon is attributed to preferential transport of ^88^Sr through the xylem, while ^86^Sr is more likely to incorporate into the phloem.

This fractionation pattern, with a different amplitude, is replicated at both sites A and B, reinforcing the hypothesis that a small mass dependent fractionation occurs during strontium assimilation from soil to fruit.

While this study focused primarily on the influence of local geology and soil on the ^87^Sr/^86^Sr signatures in cherries and cherry branches, it is important to consider potential contributions from agricultural practices. Previous research has indicated that the use of fertilizers generally does not significantly alter the Sr isotopic composition of long-lived plants, as their Sr content and isotopic signature are typically minor compared to the natural Sr pool present in the soil, which is strongly influenced by the underlying bedrock. However, data obtained from plants grown under different conditions are needed to elucidate Sr uptake behavior between different sources.

## 4. Conclusions

This study demonstrates the effectiveness of strontium isotopic ratios (^87^Sr/^86^Sr and δ^88^Sr) as reliable provenance markers for the cherry production chain. The results confirm that cherries retain the isotopic signature of bioavailable Sr from the soil, reinforcing the utility of Sr isotopes in geographical traceability studies.

The application of an external correction approach based on the ^91^Zr/^90^Zr ratio allowed for accurate mass bias correction, ensuring high reproducibility of the isotopic measurements. The observed ^87^Sr/^86^Sr ratios closely align with existing isotope maps of the Modena district, particularly for sites located near the Panaro riverbank, where Sr distribution is highly uniform.

Furthermore, the analysis of δ^88^Sr values suggests the presence of mass-dependent fractionation mechanisms during Sr uptake and translocation within the plant. This fractionation results in a progressive enrichment of ^88^Sr from soil to fruit, likely due to preferential transport processes occurring in the xylem and phloem. While the exact factors influencing this fractionation remain complex, our findings align with previous studies on Sr uptake in plants.

Overall, this study highlights the potential of Sr isotopic analysis for food authentication and geographical traceability, providing a robust tool for distinguishing cherries based on their production area. The integration of isotopic authentication systems into industrial frameworks can significantly enhance the value and credibility of agri-food products, especially those with strong territorial identity or quality labels. By providing scientifically robust proof of origin, such systems strengthen consumer trust and offer protection against fraud, ultimately supporting market competitiveness. While the analytical workflow may currently involve specialized instrumentation and trained personnel, ongoing advances in sample preparation and automation are expected to improve scalability and cost-efficiency, paving the way for broader industrial adoption. Future research should focus on refining the understanding of Sr fractionation mechanisms in plants and exploring the applicability of this approach to other food products. Furthermore, the integration of isotopic data with other traceability markers and the development of reference databases will be essential to enhance reliability and facilitate broader adoption by stakeholders.

## Figures and Tables

**Table 1:** Values of slope and intercept, with the corresponding standard deviations, and squared correlation index for the regressions of ln (^91^Zr)/(^90^Zr) vs. ln (^88^Sr)/(^86^Sr) and ln (^87^Sr)/(^86^Sr), respectively. The last column reports the theoretical slope value for both regressions.

	Slope	sd	Intercept	sd	r^2^	Theoretical Slope
ln *n*(^88^Sr)/*n*(^86^Sr)	0.488	0.004	−2.560	0.008	0.9905	0.4820
ln *n*(^87^Sr)/*n*(^86^Sr)	0.967	0.008	−1.193	0.003	0.9896	0.9571

**Table 2:** Average values of ^87^Sr/^86^Sr, ^88^Sr/^86^Sr and δ^88^Sr, and corresponding standard deviations, obtained by external correction procedure with ^91^Zr/^90^Zr as normalization isotopic ratio for the different control matrices.

	^87^Sr/^86^Sr ± S_d_			^88^Sr/^86^Sr ± S_d_			δ^88^Sr * ± S_d_		
Soil(4) ^#^	0.70875	±	0.00003	8.3758	±	0.0003	−0.33	±	0.03
Branch(3)	0.71022	±	0.00001	8.3784	±	0.0004	−0.04	±	0.04
Cherry(5)	0.70960	±	0.00006	8.3758	±	0.0003	−0.34	±	0.04

* delta (δ) values were determined by using the reference ^88^Sr/^86^Sr data equal to 8.37861. ^#^ value in parenthesis represents the number of replicated measurements.

## Data Availability

Data are contained within the article and Supplementary Materials.

## Supplementary Materials

The following supporting information can be downloaded at: https://www.mdpi.com/article/10.3390/foods14091492/s1, Table S1: Global Positioning System sampling coordinates for each producer/geographical area. Coordinates are reported in the DMS (degrees, minutes, seconds) scale. Table S2: Setup parameters used for the PM100 planetary mill. Table S3: Microwave digestion program used for the mineralization of cherry and cherry tree branch samples. Table S4: ICP/qMS instrumental setting parameters. Table S5: multi-collector ICP-MS operating parameters for the ^87^Sr/^86^Sr and ^88^Sr/^86^Sr ratio measurements. Table S6: ^87^Sr/^86^Sr mean values and associated uncertainty (u = 2 sd) for SRM NIST 987 measured in different operative conditions. Table S7: Values of ^87^Sr/^86^Sr determined for soil, tree branches, and cherries sampled from A (Castelvetro) and B (Vignola) producers. Table S8: Values of δ^88^Sr‰ determined for soil, tree branches, and cherries sampled from A (Castelvetro) and B (Vignola) producers. Figure S1: Three isotopes plot for the ln (^87^Sr/^86^Sr) vs. ln (^88^Sr/^86^Sr) measured on the NIST 987 solutions, spiked with the Zr(NO_3_)_4_ external standard, for the different working sessions. Theoretical slope for the investigated system, based on an exponential correction law, is obtained by slope ^87^Sr/^86^Sr = ln (M_87_/M_86_)/ln (M_88_/M_86_).

## Abbreviations

The following abbreviations are used in this manuscript:

PDO	Protected Designation of Origin
PGI	Protected Geographical Indication
ICP/qMS	inductively coupled plasma/quadrupolar mass spectrometer
MC-ICP/MS	multi-collector—inductively coupled plasma/mass spectrometer
