# Supplementary material for: Evaluation of the Strontium Isotope Ratios in Soil–Plant–Fruit: A Comprehensive Study on Vignola Cherry (Ciliegia di Vignola PGI)
Source: Foods. 2025 Apr 24;14(9):1492. doi: 10.3390/foods14091492 (PMC12071364; doi:10.3390/foods14091492)
Supplement: Supplementary file 1 [file foods-14-01492-s001.zip › foods-3593559-supplementary.pdf]

Table S1. Global Positioning System sampling coordinates for each producer/geographical area. Coordinates are reported in the DMS (degrees, minutes, seconds) scale.

Producer / location	Soil sampling	GPS coordinates
**A** / Castelvetro di Modena	1	44°30'23.8" N; 10°57'42.6" E
	2	44°30'24.0" N; 10°57'44.4" E
	3	44°30'22.5" N; 10°57'43.1" E
	4	44°30'21.3" N; 10°57'43.9" E
	5	44°30'21.6" N; 10°57'42.2" E
**B** / Vignola	1	44°28'38.1" N 11°00'52.1" E
	2	44°28'37.5" N 11°00'53.1" E
	3	44°28'37.7" N 11°00'54.3" E
	4	44°28'36.3" N 11°00'58.1" E
	5	44°28'35.5" N 11°00'57.8" E

Table S2. Setup parameters used for the PM100 planetary mill.

	Total time (sec)	RPM#	Reverse condition	Interval time (sec)	Pause time (sec)
1 step	90	300	On	20	10

#Revolution *per* minute

Table S3. Microwave digestion program used for the mineralization of cherry and cherry tree branches samples

	Power (Watt)	Temperature (°C)	Ramp (min)	Pressure (bar)	Time @ Temp (min)
1 step	1500	200	20:00	160	10:00

Table S4. ICP/qMS instrumental setting parameters

	He/H_2 flow (mL min^{-1})	Delay (s)	Extraction (1)	L1 (1)	L2 (1)	Focus (1)	D1 (1)	D2 (1)	PB (2)
Normal Mode	---	---	-114	-1160	-80	9.4	-43.9	-138	-1.7
3.5 KED	3.5	10	-114	-1160	-80	-10	-43.8	-138	-18
5.0 KED	5	20	-114	-1160	-80	-10	-43.8	-138	-18

	Hex. (2)	Neb. (3)	L3 (1)	Forward (4)	Hor. (5)	Vert. (5)	DA (1)	Cool (6)	Aux. (7)
Normal Mode	-1.2	0.9	-195.3	1400	106	281	-29.8	13	0.61
3.5 KED	-20	0.9	-195.3	1400	106	281	-29.8	13	0.61
5.0 KED	-20	0.9	-195.3	1400	106	281	-29.8	13	0.61

(1) Potentials, in volts, applied to a series of lenses that allow to focus the ion beam.
(2) Potentials, in volts, applied respectively to the quadrupole mass analyzer (Pole Bias) and the hexapole collision cell (Hexapole Bias).
(3) Ar flow in L/min, which allows the formation of the sample aerosol and carries the droplets in the plasma torch
(4) Power (in watts) of RF energy emitted by the generator for the plasma formation.
(5) Position of the plasma torch, in mm.
(6) Plasma cooling gas in L/min.
(7) Ar flow in L/min, which contributes to the plasma formation.

Table S5. Multi collector ICP-MS operating parameters for the $^{87}Sr/^{86}Sr$ and $^{88}Sr/^{86}Sr$ ratio measurements

**Parameter**	**Sr wet plasma**	**Sr dry plasma**
RF power (W)	1245	1245
Gas flow rate (L min^{-1})	Sample gas: 0.95 – 1.05<br>Auxiliary gas: 1.00<br>Cooling gas: 16	Sample gas: 0.95 – 1.05<br>Auxiliary gas: 1.00<br>Cooling gas: 16
Sample/Skimmer cone	Ni	Ni
Injector	Sapphire	Sapphire
Spray chamber	Quartz Cyclonic	APEX-IR
Nebulizer	PFA micro flow self-aspirating	HT-PFA micro flow self-aspirating
Faraday Cup configuration - Amplifiers	L4 – ^{83}Kr - 10^{12}<br>L3 – ---<br>L2 – ^{85}Rb - 10^{12}<br>L1 – ^{86}Sr – 10^{11}<br>C – ^{87}Sr – 10^{11}<br>H1 – ^{88}Sr – 10^{11}<br>H2 – ^{90}Zr – 10^{11}<br>H3 – ^{91}Zr – 10^{11}<br>H4 – ^{92}Zr – 10^{11}	L4 – ^{83}Kr - 10^{12}<br>L3 – ---<br>L2 – ^{85}Rb - 10^{12}<br>L1 – ^{86}Sr – 10^{11}<br>C – ^{87}Sr – 10^{11}<br>H1 – ^{88}Sr – 10^{11}<br>H2 – ^{90}Zr – 10^{11}<br>H3 – ^{91}Zr – 10^{11}<br>H4 – ^{92}Zr – 10^{11}
Control Cup for peak center	C – ^{87}Sr – 10^{11}	C – ^{87}Sr – 10^{11}
Mass analyzer pressure (mbar)	$< 10^{-8}$	$< 10^{-8}$
Background/baseline determination	4% (v/v) HNO_3	4% (v/v) HNO_3
Sample uptake rate (μL min^{-1})	100	100
Uptake time (s)	150	150
Idle time (s)	10	10
Washing time (s)	100	100
Number of blocks	1	1
Number of cycles	100	100
Integration time (s)	8.839	8.839
APEX-IR heather temperature (^{0}C)	---	100
APEX-IR chiller temperature (^{0}C)	---	2
APEX-IR solution Uptake (μL min^{-1})	---	100
Sensibility (V ppm^{-1})	> 70 for ^{88}Sr	> 700 for ^{88}Sr
Sensibility (V ppm^{-1})	> 45 for ^{90}Zr	> 400 for ^{90}Zr

Table S6. $^{87}Sr/^{86}Sr$ mean values and associated uncertainty (u = 2sd) for SRM NIST 987 measured in different operative conditions

NIST 987	Conventional nebulization (200 μg Kg^{-1}) n = 142	APEX IR (10 μg Kg^{-1}) n = 23	APEX IR (20 μg Kg^{-1}) n = 17
**Mean ± u**	0.71027 ± 0.00003	0.71028 ± 0.00006	0.71027 ± 0.00003

Table S7. Values of $^{87}Sr/^{86}Sr$ determined for soil, tree branches and cherries sampled from A (Castelvetro) and B (Vignola) producers.

Soil_(A)	Shoots_(A)	Cherry_(A)	Soil_(B)	Shoots_(B)	Cherry_(B)	Cherry_(Tot)	Cherry_(Tot)
0.70987	0.70989	0.70987	0.70892	0.70898	0.70875	0.70916	0.70911
0.70988	0.70991	0.70951	0.70882	0.70896	0.70917	0.70911	0.70899
0.70978	0.70988	0.70899	0.70888	0.70909	0.70887	0.70964	0.70923
0.70985	0.71024	0.70906	0.70899	0.70896	0.70880	0.70845	0.70921
0.71018	0.71005	0.71016	0.70891	0.70896	0.70888	0.70895	0.70926
		0.70973			0.70905	0.70892	0.70922
		0.70992			0.70872	0.70987	0.70986
						0.70951	0.70872
						0.70899	0.70959
						0.70876	0.70905
						0.71016	0.70866
						0.70973	0.70900
						0.70992	0.70905
						0.70912	0.70929
						0.70901	0.70867
						0.70919	0.70848
						0.70984	0.70909
						0.70976	0.70884
						0.70996	0.70958
						0.70980	0.70943
						0.70876	0.70937
						0.70878	0.70934
						0.70924	0.70949
						0.70924	0.70888
						0.70888	0.70905
						0.70964	0.70898
						0.70977	0.70922
						0.70954	0.70935
						0.70941	0.70916
						0.71011	0.70939
						0.70890	0.70887
						0.70899	0.70905
						0.70877	0.70993
						0.70946	0.70893
						0.70902	0.70979
						0.70937	0.70970
						0.70926	0.70987
						0.70834	0.70875

0.70888	0.70917
0.70887	0.70887
0.70949	0.70880
0.70939	0.70981
0.70923	0.70922
0.70987	0.70921
0.70879	
0.70859	
0.70871	
0.70974	

Table S8. Values of d^{88}Sr (‰) determined for soil, tree branches and cherries sampled from A (Castelvetro) and B (Vignola) producers.

Soil_(A)	Shoots_(A)	Cherry_(A)	Soil_(B)	Shoots_(B)	Cherry_(B)	Cherry_(Tot)	Cherry_(Tot)
-0.08	-0.06	-0.09	-0.42	-0.04	0.08	-0.40	-0.20
-0.18	-0.03	-0.23	-0.36	-0.07	-0.13	-0.83	-0.19
-0.32	-0.08	0.14	-0.29	-0.11	0.01	-0.31	-0.10
-0.22	-0.07	-0.12	-0.24	-0.10	0.07	-0.71	-0.47
-0.24	-0.04	0.05	-0.36	-0.15	0.16	-0.28	-0.87
		0.17			0.14	-0.05	-0.21
					0.07	-0.09	0.13
						-0.76	0.07
						-0.23	-0.73
						0.14	-0.88
						-0.12	-0.49
						0.05	-0.31
						0.17	0.01
						-0.43	0.07
						-0.62	0.16
						0.01	0.11
						-0.39	0.11
						-0.42	-0.20
						-0.16	0.24
						-0.35	-0.07
						-0.34	-0.10
						-0.04	0.01
						0.05	-0.18
						-0.23	0.14
						-0.26	-0.29
						-0.26	-0.02
						-0.77	-0.17
						-0.36	-0.27
						-0.39	-0.37
						-0.33	-0.93
						-0.31	-0.31
						0.15	-0.90
						-0.22	-0.40
						0.15	0.20
						0.15	-0.42
						-0.99	-0.23
						-0.47	0.07
						0.07	0.08
						-0.13	
						-0.06	

-0.16
-0.02
0.18
-0.33
-0.16
-0.69
0.09
-0.01
-0.42
-0.30
-0.38

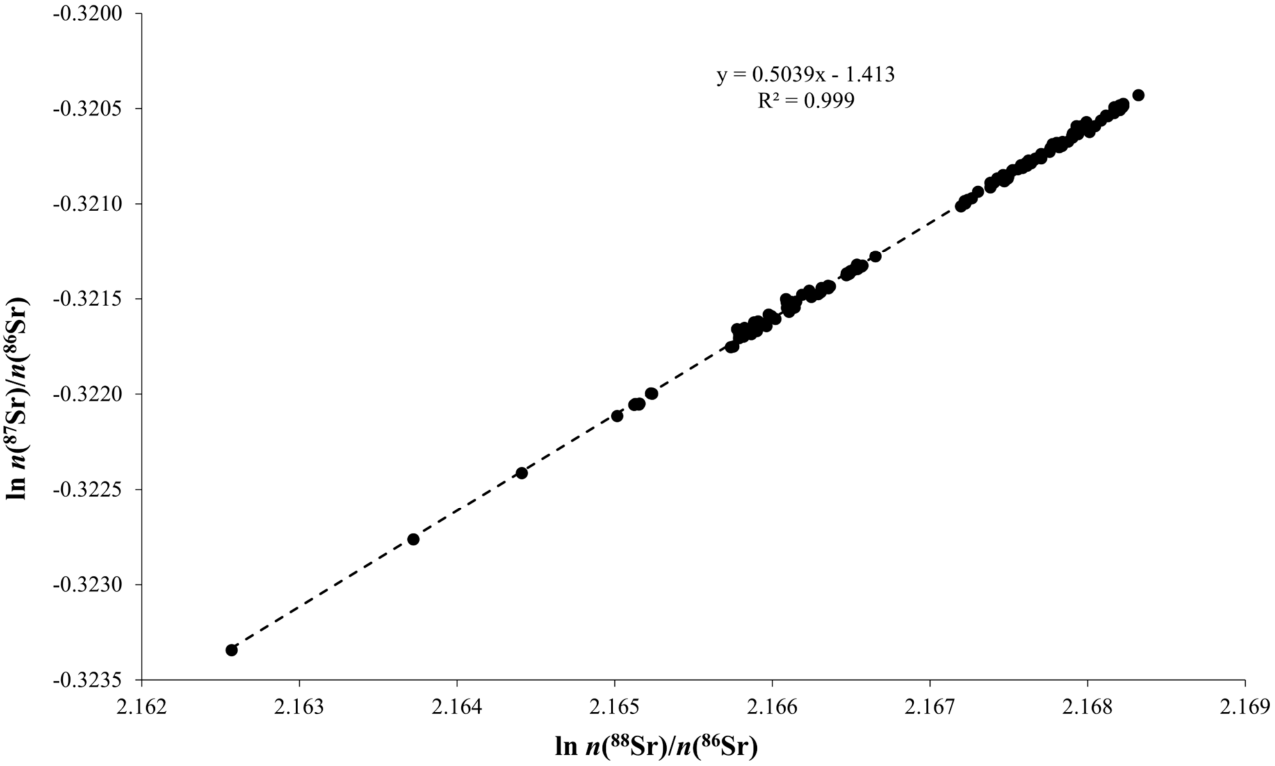

Figure S1. Three isotopes plot for the ln(^{87}Sr/^{86}Sr) *vs.* ln(^{88}Sr/^{86}Sr) measured on the NIST 987 solutions, spiked with the $Zr(NO_3)_4$ external standard, for the different working sessions. Theoretical slope for the investigated system, based on an exponential correction law, is obtained by: slope ^{87}Sr/^{86}Sr = ln(M_{87}/M_{86}) / ln(M_{88}/M_{86}).
